# Synthesis of a Carrageenan–Iron Complex and Its Effect on Flame Retardancy and Smoke Suppression for Waterborne Epoxy

**DOI:** 10.3390/polym11101677

**Published:** 2019-10-14

**Authors:** Na Wang, Haiwei Teng, Xinyu Zhang, Jing Zhang, Long Li, Jing Zhang, Qinghong Fang

**Affiliations:** 1 SinO–Spanish Advanced Materials Institute, Shenyang University of Chemical Technology, Shenyang 110142, China; 18842411721@163.com (H.T.); zhangjingcszx@syuct.edu.cn (J.Z.); lilong@syuct.edu.cn (L.L.); qinghongfang@syuct.edu.cn (Q.F.); 2 Liaoning Provincial Key Laboratory of Rubber & Elastomer, Shenyang 110142, China; 3 Advanced Manufacturing Institute of Polymer Industry (AMIPI), Shenyang University of Chemical Technology, Shenyang 110142, China; 4 IMDEA Materials Institute, C/Eric Kandel, 2, 28906 Getafe, Madrid, Spain; jing.zhang@imdea.org

**Keywords:** flame retardancy, waterborne epoxy, k-carrageenan, APP

## Abstract

A k-carrageenan–iron complex (KC–Fe) was synthesized by complexation between degraded KC and FeCl_3_. Furthermore, KC–Fe and ammonium polyphosphate (APP) were simultaneously added into waterborne epoxy (EP) to improve its flame retardancy and smoke suppression performance. The structure and properties of KC–Fe were assessed using Fourier transform infrared spectroscopy (FTIR), ultraviolet (UV) spectroscopy, thermo gravimetric analysis (TGA), and X-ray powder diffraction analysis (XRD). The analysis showed that KC–Fe was successfully synthesized and exhibited good thermal properties with a 49% char residue at 800 °C. The enhanced flame retardancy and smoke suppression performance of waterborne epoxy were evaluated using a limiting oxygen index (LOI) and UL-94. Moreover, the flame retardancy of waterborne epoxy coated on a steel plate was also investigated using cone calorimetry. The results showed that the flame-retardant waterborne epoxy blend exhibited the best flame retardancy when the mass ratio of APP and KC–Fe was 2:1. The total heat release (THR) and total smoke production (TSP) was decreased by 44% and 45%, respectively, which indicated good fire safety performance and smoke suppression properties. Analysis of the residual char using FTIR, SEM, and elemental analysis (EDS) indicated that the action of KC–Fe was promoted by the presence of APP. The formation of a dense thermal stable char layer from an intumescent coating was essential to protect the underlying materials.

## 1. Introduction

Waterborne epoxy has been used in various fields, such as adhesives, surface coatings, and composites, because of its superior chemical and physical properties [1,2,3]. However, the flammability of waterborne epoxy limits its application in some areas [4,5,6,7]. Moreover, waterborne epoxy contains a high level of aromatic ring structures, which can generate a large amount of heat and toxic fumes when undergoing combustion. The inhalation of lethal smoke is a major cause of death when a large fire occurs [8,9,10,11]. Therefore, it is of great practical significance to improve the flame retardancy of waterborne epoxy and to reduce the amount of released smoke from the burning of waterborne epoxy.

In recent years, it has been found that the addition of metal oxides and flame-retardants simultaneously into polymer composites can not only significantly improve the flame-retardant properties of the composites, but also improve their smoke suppression properties [12,13,14,15,16]. Recently, it was reported that Fe_2_O_3_ readily forms a stable Fe–O–P cross-linked network structure with phosphoric acid, thereby increasing the char residue for a flame-retardant coating at high a temperature. Fe_2_O_3_ promoted the transformation of the char layer with high graphitization, thereby effectively improving the flame-retardant properties of the coating [17]. The preparation of intumescent flame-retardant materials using molybdenum trioxide and antimony trioxide as promotes has been reported [18,19,20]. Studies have shown that the presence of molybdenum trioxide delayed the ignition time of flame-retardant coatings due to the cross-linking reaction of small molecules during combustion. The molybdenum trioxide and antimony trioxide further exhibit a catalytic effect that promotes the formation of a more dense foamed char layer, thereby reducing the volatilization of combustibles and achieving the suppression of smoke. However, the use of metal oxides in combination with flame retardants is not sufficient to achieve a highly efficient cooperative effect for polymers [21,22,23,24]. Instead, a metal-species-modified material with an in-situ method has been reported as an effective way of providing polymers with enhanced flame retardancy [25,26].

In the preliminary work of our group, it was found that the bio-based material k-carrageenan was an excellent flame retardant [27,28]. Therefore, in order to further improve its smoke suppression performance, the k-carrageenan–iron complex (KC–Fe) was obtained by grafting a carrageenan molecule with the transition metal element Fe through a complexation reaction. The structure and thermal stability of the KC–Fe was characterized using Fourier transform infrared spectroscopy (FTIR), ultraviolet spectroscopy (UV), and thermogravimetric analysis (TGA). The crystal plane structure of the KC–Fe after combustion was characterized using X-ray powder diffraction (XRD). The effects of KC–Fe and APP on the flame retardancy, smoke suppression performance, and thermal decomposition behavior of waterborne epoxy (EP) were studied under different mixing ratios. The corresponding mechanism study in the condensed phase was also applied to explain the formation of thermal stable residue and compact char residue, which is crucial in the flame-retardant coating system.

## 2. Materials and Methods

### 2.1. Materials

Degraded k-carrageenan was made by our laboratory. Anhydrous ethanol and potassium chloride were obtained from Tianjin Yongda Chemical Reagent Co., Ltd. (Tianjin, China). Hydrogen peroxide was obtained from Liaoning Jiacheng Fine Chemicals Co., Ltd. (Fuxin, China). Ferric chloride and sodium citrate were obtained from Tianjin Damao Chemical Reagent Factory (Tianjin, China). Sodium hydroxide was obtained from Tianjin Ruijinte Chemical Co., Ltd. (Tianjin, China). Waterborne epoxy resin was obtained from Hexion Specialty Chemicals, Inc. (Columbus, Ohio, USA).

### 2.2. Methods

#### 2.2.1. Synthesis of KC–Fe

**Figure 1:**
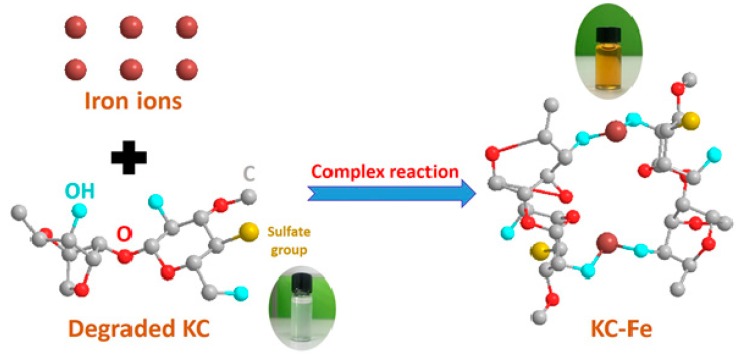
Preparation of the k-carrageenan–iron complex (KC–Fe) sample.

The reaction process of KC–Fe is shown in Figure 1. A total of 2.0 g of degraded KC, 0.5 g of sodium citrate, and 60 mL of distilled water were added to a three-necked flask, and the solution was stirred in a 70 °C water bath until completely dissolved. Next, 2 mol/L of ferric chloride solution and a 20% sodium hydroxide solution were slowly added dropwise. The pH of the control solution was in the range of 8.0–8.5. When a brown-red insoluble precipitate appeared in the solution, the further addition of ferric chloride and sodium hydroxide solution was immediately stopped. We continued to stir the solution in a hot water bath for 1 h. After the reaction solution was cooled, centrifuge was applied at 3500 rpm for 15 min to separate materials. Triple volumes of absolute ethanol (98%) were added into the upper layer of centrifuged liquid with the appearance of a dark reddish brown liquid. Afterward, a reddish brown precipitate appeared in the solution. After completing the precipitation, the solution was centrifuged, and the precipitate was further washed with 95% ethanol and absolute ethanol, and dried under vacuum to obtain KC–Fe.

#### 2.2.2. Preparation of Flame-Retardant EP

Based on extensive research by our lab on flame retardants in polymers, this paper mainly explores the effect of different mass ratios of KC–Fe and APP on the flame retardancy and smoke suppression performance of waterborne epoxy resin with the total weight fraction at 30 wt%. A series of flame-retardant coatings with different fillers were prepared; the formulation is listed in Table 1. Taking the flame-retardant coating with 30 wt% KC–Fe as an example, KC–Fe (4.50 g) was first blended with waterborne epoxy resin (7.50 g) using a pearl mill for 30 min. The curing agent (3.00 g) and deionized water were added into the compound and stirred at room temperature for 30 min. The coating was scraped onto one side of a 100 × 100 × 1 mm^3^ steel plate, and the sample was cured at room temperature for one week before the test. Similarly, other flame-retardant coatings in Table 1 were prepared in the same way.

#### 2.2.3. Characterization

Fourier transform infrared spectroscopy (FTIR) was recorded on a Nicolet MNGNA-IR560 (Artisan Technology Group, Austin, TX, USA) with a transition mode and a wave-number range between 400 and 4000 cm^−1^. The ultraviolet spectrum was recorded on a UV-2550 UV-Vis Spectrophotometer (Shimadzu Corporation, Tokyo, Japan). The 0.05% concentration of KC, FeCl3, and KC–Fe aqueous solutions were prepared for the UV test with a spectral region wave-number range from 200 to 700 nm. Thermogravimetric analyses (TGA) were tested on an STA 449C thermal analyzer (Selb, Germany) from 40 to 800 °C at a heating rate of 10 °C/min under a nitrogen atmosphere. X-ray diffraction patterns (XRD) of the residue of KC–Fe, after burning at 500 °C, were recorded on a D8 Advance X-ray diffractometer (Bruker, Germany) with Cu Kα radiation (λ = 0.154) and a scanning speed of 5°/min. Limited oxygen index (LOI) data were obtained using an oxygen index instrument (JF-3) (Jiangning Analysis Instrument Company, Nanjing, China) according to the GB/T 2406-2009 standard. The dimensions of the specimens were 126 × 6.5 × 3 mm^3^. The vertical burning test was carried out on a CZF-3-type instrument (Jiangning Analysis Instrument Company, Nanjing, China) according to the ASTM D3801-2010 standard. The dimensions of the specimens were 130 × 13 × 3 mm^3^. The cone calorimeter tests were carried out on a fire testing technology (FTT, England, UK) cone calorimeter. The specimens were irradiated at a heat flux of 50 kW/m^2^ according to ISO 5660-1 standard procedures. The SEM images of the burned samples were carried out on an SEM JEOL JSM-6360LV (Japan), equipped with energy dispersive X-ray spectroscopy (EDS). The burnt samples from the LOI analysis were used for testing. The complete burning of the samples was ensured.

## 3. Results

### 3.1. Characterization of KC–Fe

**Figure 2:**
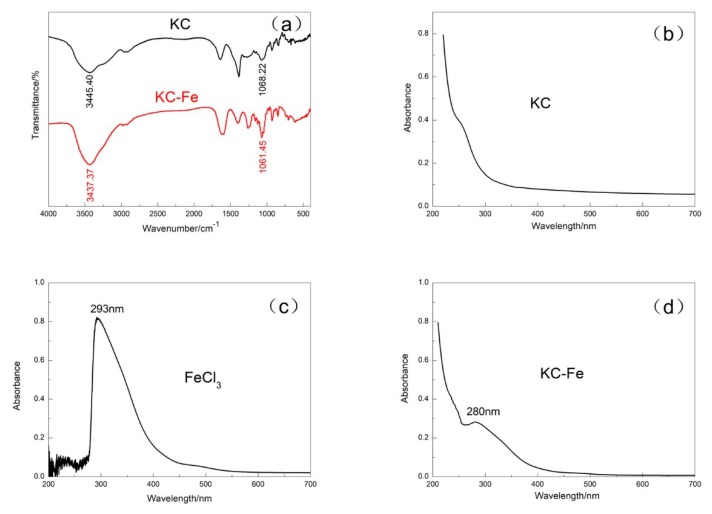
(**a**) Fourier transform infrared spectroscopy (FTIR) spectra of KC and KC–Fe; the UV spectra of (**b**) KC, (**c**) FeCl_3_, and (**d**) KC–Fe.

After modifying KC with iron ions, the product became a reddish brown powder, which was different to the white color of the original KC. As shown in Figure 2a, the hydroxyl groups from the IR spectrum of the KC sample exhibited a peak at 3450.40 cm^−1^. However, the hydroxyl group of the Fe–KC sample shifted to a lower wavenumber (3437.37 cm^−1^). In addition, the stretching vibration of C–OH also changed from the original 1068.22 to 1061.45 cm^−1^, which indicated a coordination reaction between the hydroxyl group on KC and the Fe ion. The typical characteristic peaks resulting from the KC were observed in both KC and KC–Fe samples, including the symmetric stretching vibration peak of O–S–O at 1262.21 cm^−1^, the stretching vibration peak of C–O–S at 841.03 cm^−1^, and the stretching vibration peak of C–O–C at 930.62 cm^−1^. This demonstrated that the structure of KC was not destroyed during the synthesis process. The UV absorption results for KC, FeCl_3_, and KC–Fe samples further confirm the above conclusion. FeCl_3_ showed a characteristic UV absorption peak at 293 nm, but no characteristic absorption peak appeared for the KC sample. This is consistent with the general structural characteristics of carbohydrates. A significant blue shift (280 nm) was observed on the UV spectrum for the KC–Fe sample. This is due to the coordination reaction between the hydroxyl groups contained in the carrageenan with Fe^3+^. The degree of delocalization of electrons in the whole molecule increased, and the energy required for the electron transition decreased. Combined with the FTIR and UV results, it can be concluded that the carrageenan reacts with Fe^3+^ through coordination bonds [29].

**Figure 3:**
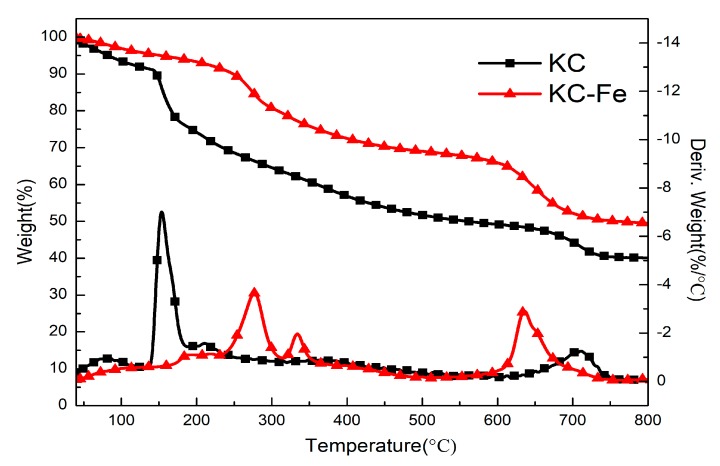
Thermogravimetric analysis and DTG curve of KC and KC–Fe under N_2_. (The top two lines belong to the left y-axis; the bottom two lines belong to the right y-axis).

Figure 3 presents the TG and DTG curves of KC and KC–Fe under N_2_. As shown in the figure, the mass loss of KC before 150 °C was due to the thermal evaporation of intramolecular bound water [27]. KC decomposed in two steps between 150 and 800 °C and the two peaks related to the maximum degradation rate exhibited on the DTG curve appeared at 154 and 708 °C, respectively. The decomposition at 154 °C was mainly caused by the decomposition of glycogenic bonds, by the six-membered ring, and by decarboxylation, leading to the release of small molecular compounds such as H_2_O, CO_2_, CO, etc. The second maximum degradation temperature appeared at 708 °C, which was due to decomposition of an unstable char layer [30]. The final residual char mass was 42% at 800 °C. The complex KC–Fe exhibited a higher thermal stability. The first decomposition step was significantly increased to around 277 °C, and the weight loss at this temperature was also reduced [31]. The weight loss at a higher temperature (633 °C) was due to the formation of a barrier char layer. This barrier char hindered the release of gas from the oxidation of the internal carbide, indicating a potential flame retardancy property. With the continuous accumulation of internal gases and the decomposition of surface carbides, the gas broke through the barrier layer, and the weight loss was observed at this temperature. The final amount of char residue (49%) was higher than that of KC (42%).

**Figure 4:**
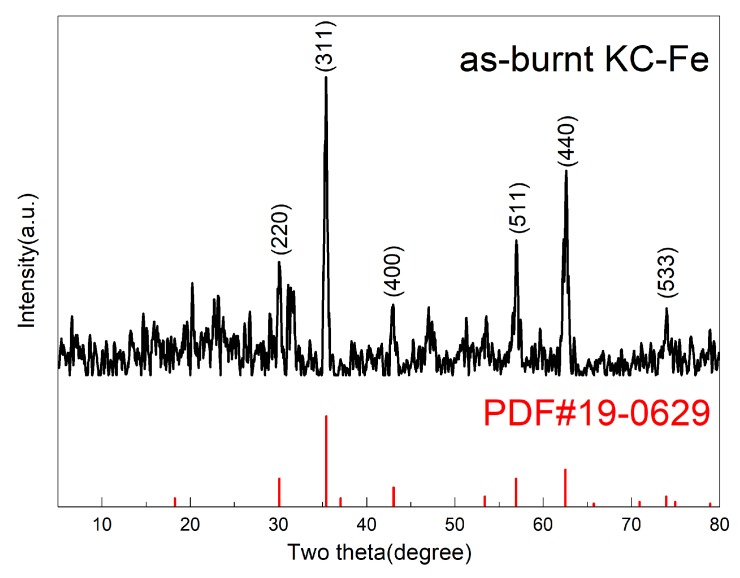
X-ray powder diffraction analysis (XRD) pattern of residual substances after complete combustion of KC–Fe.

In order to determine the structural composition of KC–Fe after combustion, XRD analysis was carried out for the residual material of KC–Fe after being treated in a muffle furnace at 500 °C. As shown in Figure 4, the diffraction peaks located at 30.10°, 35.42°, 43.05°, 56.94°, 62.52°, and 73.95° corresponded to Fe_3_O_4_ crystal faces (220), (311), (400), (511), (440), and (533), according to the standard PDF #19-0629 card [31]. Based on this result, it is clear that iron oxides, which exhibit a good catalytic charring effect by changing flammable small molecules into macromolecules, can be formed from KC–Fe. This may provide polymers with enhanced smoke suppression properties.

### 3.2. Thermal Stability of Flame-Retardant EP

**Figure 5:**
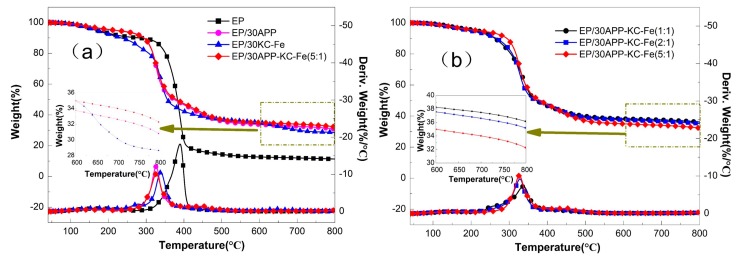
Thermogravimetric analysis and DTG curves of FR-EP under N_2_. (**a**) The top four lines belong to the left y-axis; the bottom four lines belong to the right y-axis; (**b**) the top three lines belong to the left y-axis; and the bottom three lines belong to the right y-axis.

The thermal stability and char forming ability of the flame-retardant EP were investigated by TGA, and the data are shown in Table 2 and Figure 5. In Figure 5a, the initial decomposition temperature of all flame-retardant EP was lower than that of pure EP [32], especially for the EP/30KC–Fe components. This is due to the catalytic decomposition effect of a metal-based flame retardant [33]. When the temperature was raised to 390 °C, the flame-retardant EP exhibited improved stability without significant weight loss compared with pure EP. This was ascribed to the decomposition of the flame retardant to produce a stable char layer structure, thereby protecting the matrix from thermal decomposition. With the addition of APP alone, the char residue at 800 °C increased significantly from 11.3% to 30.6%. It can be seen from Figure 5a that the maximum degradation rate of EP/30APP was greatly reduced compared with pure EP, which was due to the partial decomposition of APP. The formation of phosphoric acid and polyphosphate acid promoted the formation of a more stable char layer structure and inhibited further decomposition of the matrix [34]. When KC–Fe was added alone, the thermal stability of EP was enhanced after 387 °C, compared with pure EP. However, there was a slight weight loss at 632 °C, indicating that the char layer formed by metal catalysis was unstable at high temperatures. However, the amount of char residue at 800 °C was still improved, compared to pure EP, reaching 28.6%. With the addition of APP and KC–Fe with a mass ratio of 5:1, the amount of char residue of EP/30APP–KC–Fe (5:1) at 800 °C reached 32.1%, which was higher than it was with the addition of the single flame retardants separately. This result indicates that APP and KC–Fe have significant synergistic charcoal effects. The KC–Fe-derived iron oxides can effectively catalyze the thermal generation of APP to produce metaphosphoric acid and polyphosphoric acid due to the principle of free radical trapping. Thereby, the EP matrix and the KC–Fe can form a char layer structure through an esterification reaction. On the other hand, iron oxides can also adsorb small molecules with poor thermal stability to form macromolecules with improved thermal stability, thereby forming a char layer structure that can effectively isolate the matrix from oxygen and flame contact stability [35]. Furthermore, the optimal ratio of APP to KC–Fe was explored, and all three EP composites with different ratios showed an enhanced char residue and a reduced maximum degradation rate. The enhanced thermal stable char residue can protect the underlying steel when it is used as a flame-retardant coating to insulate the transfer of heat.

### 3.3. Intrinsic Fire Behavior of Waterborne Epoxy

In order to evaluate the intrinsic flame retardancy of the waterborne epoxy, the prepared waterborne epoxy and its composites were tested by LOI and UL-94, and the relevant data are given in Table 3. As can be seen from Table 3, pure EP showed a high flammability level. During the test, a vigorous flame, accompanied with the generation of smoke generation and droplet dripping, was observed. Its LOI value was only 18.6% and did not achieve any rating in the UL-94 test. For the EP/30APP sample, the LOI value increased rapidly, reaching 30.2%. This is because the APP was heated to generate non-combustible gas to dilute the surrounding oxygen, thereby increasing the LOI value of the sample. After adding KC–Fe alone, the flame retardancy was not improved, and the LOI value was only 22.8%, but the amount of smoke was significantly reduced during the combustion of the sample. By simultaneously filling APP and KC–Fe (1:1) in EP, the LOI value was increased, and the vertical burning test was also V-2. The above results show that APP and KC–Fe exhibited an excellent synergistic effect to improve the flame-retardant properties of EP. By adjusting the ratio of APP and KC–Fe to 2/1 and 5/1, both EP composites showed improved flame retardancy, passing the UL-94 V-1 rating with LOI values of 29.5 and 27.3, respectively. 

In order to further study the effects of the addition of KC–Fe and APP on the flame-retardant and the fire safety of the coating material, we performed a cone calorimetry test on the EP coating containing KC–Fe and APP on the steel plate. The cone test was a bench scale fire test that could simulate the actual combustion of the material in the fire. The test data obtained could be used to evaluate the combustion behavior of the material in the fire. Many important combustion parameters, such as peak heat release rate (pHRR), total heat release (THR), smoke release rate (SPR), total smoke production (TSP), carbon monoxide production (COP), and carbon dioxide production (CO_2_P), can be used to determine the potential fire hazard of polymer materials. 

**Figure 6:**
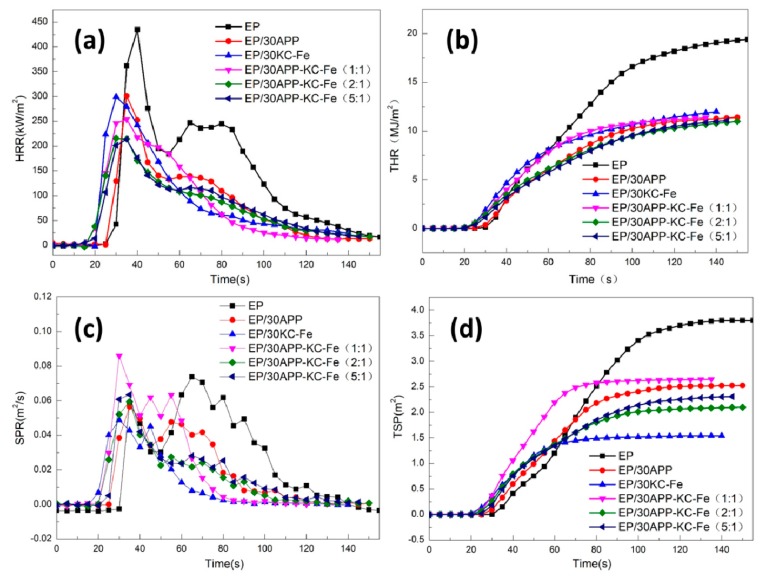
(**a**) The heat release rate (HRR) curves of the pure waterborne epoxy (EP) and FR-EP; (**b**) THR curves of the pure waterborne epoxy (EP) and FR-EP; (**c**) SPR curves of the pure waterborne epoxy (EP) and FR-EP; and (**d**) TSP curves of the pure waterborne epoxy (EP) and FR-EP.

The heat release rate (HRR), especially the pHRR value, has been shown to be an important parameter for assessing the fire safety of materials. The HRR and THR data for pure EP and its composites are shown in Figure 6a,b. The pure EP coating quickly burned and released heat after ignition. The pHRR value quickly reached 434.5 kW/m^2^, and the THR value was as high as 19.3 MJ/m^2^. When adding either APP or KC into EP coating, the pHRR value and the THR value of the flame-retardant coating were reduced, compared with that of pure EP. The pHRR and THR value of the EP/30APP sample decreased by 31% and 41%, respectively. A 31% decrease in pHRR and a 38% decrease in THR were obtained for the EP/30KC sample. This indicated that the addition of APP or KC–Fe could improve the flame retardancy of the coating. With the addition of APP and KC–Fe simultaneously in EP, the THR and pHRR values of all EP/30APP-KC–Fe composites further decreased significantly, compared with EP, EP/30APP and EP/30KC–Fe, indicating that APP and KC–Fe have synergistic effects. The influence of the ratio between APP and KC–Fe on the flame retardancy of EP was shown in Figure 6a,b. The sample EP/30APP-KC–Fe (2:1) exhibited the best property with a 51% (213.2 kW/m^2^) and 44% (10.9 MJ/m^2^) decrease in pHRR and THR, respectively (as shown in Table 4). The results showed that the EP/30APP–KC–Fe (2:1) composite had excellent flame-retardant properties. The significant decrease in pHRR and THR of EP/30APP–KC–Fe (2:1) composites was due to the synergistic flame-retardant effect of APP and KC–Fe. APP was thermally decomposed to produce non-combustible gas, and the oxygen concentration in the combustion zone was diluted. Meanwhile, the char layer was formed with cross-linked polyphosphoric acid and metaphosphoric acid. KC–Fe-derived iron oxide may adsorb flammable volatiles and promote the formation of a dense protective char layer [36,37], which acted as a barrier to the external heat source.

In addition, the smoke release rate (SPR) curve and the total smoke production (TSP) curve of the EP and EP/APP–KC–Fe composites are shown in Figure 6c,d. With the addition of KC–Fe, the TSP and SPR of the EP/30KC–Fe sample were significantly reduced by 60% and 34%, respectively. The reason for the significant reduction in SPR and TSP is attributed to the iron oxide derived from KC–Fe. Iron oxide has been reported to have an excellent catalytic charring effect to promote the formation of a dense char layer by changing flammable small molecules into macromolecular char, thus further preventing the flammable gas from diffusing into the air. This indicated the excellent flame-retardant and smoke suppression properties of the KC–Fe-based waterborne epoxy. Therefore, the EP coating with the addition of APP and KC–Fe simultaneously exhibited a lower TSP and COP value, compared with pure EP, due to the existence of KC–Fe, which also formed a compact char layer in the condensed phase.

**Figure 7:**
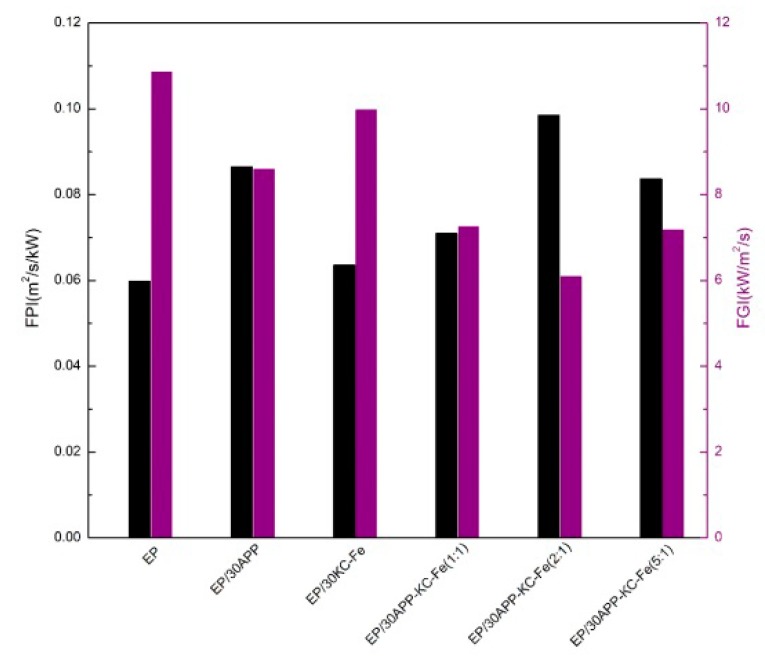
Fire performance index (FPI) (black columns) and FGI (purple columns) values for the pure waterborne epoxy (EP) and FR-EP at a flux of 50 kW/m^2^.

Fire performance index (FPI, the ratio of sample ignition time to heat release rate peak) and the fire development index (FGI, the ratio of the pHRR of a sample to the time required to reach that peak) are important parameters to evaluate the fire behavior of polymers. As shown in Figure 7, the pure EP coating has the lowest FPI value (0.0598) and the highest FGI value (10.8624), which indicated that the pure EP coating will be ignited in a very short time in the fire. The optimal sample EP/30APP–KC–Fe (2:1) exhibited the highest FPI value (0.0984) and the lowest FGI value (6.0937). Therefore, the EP intumescent coating showed the lowest fire risk and the highest safety level with the addition of APP and KC–Fe.

### 3.4. Analysis of Char Residue

**Figure 8:**
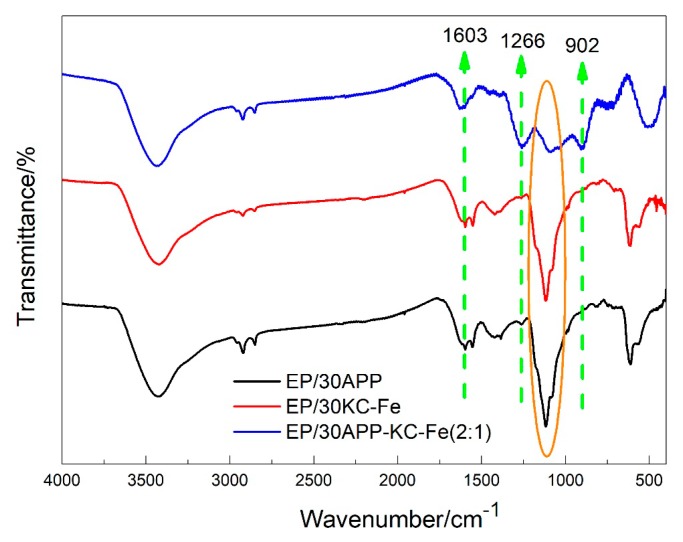
FTIR spectra of char from EP and FR-EP.

In order to study the flame-retardant mechanism of the EP and the synergistic effect of the APP and KC–Fe interaction, the residual char produced after the LOI combustion experiment was analyzed via an FTIR test. It can be seen from Figure 8 that all combustion products have an absorption peak at 3400 cm^−1^, indicating that the compound containing hydroxyl groups (–OH) was produced during combustion [38]. All three samples showed vibration absorption peaks of the aromatic fused ring skeleton at 1603 cm^−1^, and this structure can effectively improve the thermal stability of the flame-retardant EP. However, the two peaks only appeared for the EP/30APP-KC–Fe (2:1) sample at 1266 and 902 cm^−1^, corresponding to the P=O bond and P–O bond from polyphosphoric acid produced by the decomposition of APP. This char layer structure can effectively enhance the barrier properties and high temperature resistance of the char layer. In addition, the intensity of the peaks at 1114 cm^−1^, which is attributed to the C–O stretching vibration peak from the EP/30APP and EP/30KC–Fe samples, was significantly greater than the EP/30APP–KC–Fe (2:1) sample. This indicated that the aliphatic alcohols were formed in the combustion process. The reduced peak at 1114 cm^-1^ resulted from the oxidation of incompletely burned substances by iron oxides.

**Figure 9:**
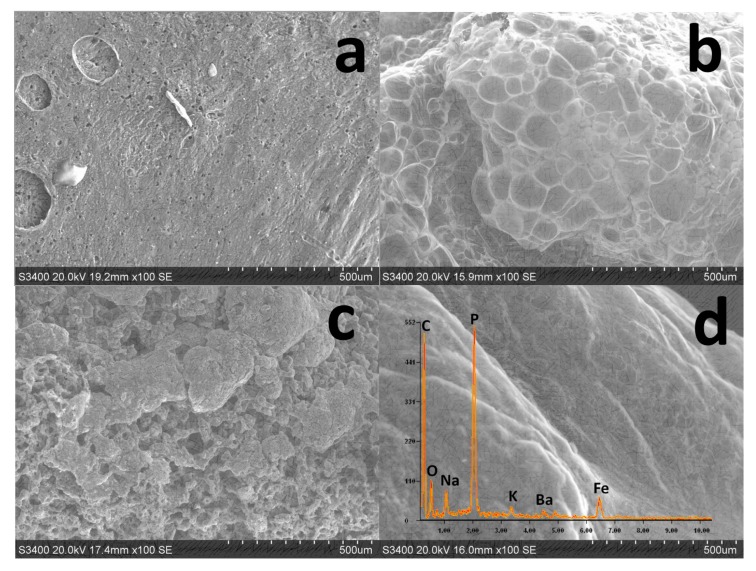
Scanning electron microscopy (SEM) micrographs of chars after LOI test. (**a**) EP; (**b**) EP/30APP; (**c**) EP/30KC–Fe; and (**d**) EP/30APP–KC–Fe (2:1).

The structure and morphology of the char residue from the LOI test was analyzed by SEM and EDS, as shown in Figure 9. Pure EP exhibited a droplet structure after combustion, which allowed for the transmission of heat and oxygen easily. With the presence of only APP in EP, a continuously expandable and porous residual char can be observed. This is due to the thermal decomposition of APP accompanied by the release of polyphosphoric acid, which promoted the formation of a continuously expanding char layer [39,40]. The release of volatiles broke through the layer, leading to the porous surface structure. With the addition of only KC–Fe into the EP, a loose char layer structure with a porous structure was formed, which was not sufficient to protect the underlying substrate from the flame. Interestingly, when APP and KC–Fe were simultaneously added to the EP at a 2:1 addition ratio, the synergistic effect of the two materials contributed to the formation of a more compact and continuously expanded char layer structure with the ability to suppress heat and oxygen. Furthermore, the elemental composition of the char layer analyzed by EDS showed the existence of Fe elements. This is due to the formation of iron oxides derived from KC–Fe, which is consistent with the XRD results of combusted KC–Fe. The catalytic effect of metal oxide may promote the formation of a char layer with a compact structure, according to the FTIR analysis for the char residue. The barrier effect of the char layer can slow down further char loss and enhance the stability of the char layer, and this is consistent with the thermogravimetric test results of the flame-retardant EP. This test indicated that KC–Fe was thermally decomposed in the condensed phase and that the iron oxide formed by thermal decomposition was stored in the residual char of the flame-retardant EP. The iron oxide can adsorb and catalyze the combustion of small molecular substances to form a stable char layer structure, thereby achieving a smoke suppressing effect [12,41].

## 4. Conclusions

In this study, the bio-based flame-retardant carrageenan–iron complex was successfully synthesized through degraded k-carrageenan with FeCl_3_. The TGA data showed that Fe-modified KC can improve the high temperature char forming performance and thermal stability of KC–Fe. Next, KC–Fe and APP were simultaneously added to EP to prepare EP/APP–KC–Fe flame-retardant samples. Various fire tests, including LOI, UL-94, and the cone calorimeter test, demonstrated that the EP/30APP–KC–Fe (2:1) sample can achieve the best intrinsic flame-retardant and smoke suppression properties with a high LOI value (29.5%), a UL-94 V-1 rating, and a significant decrease in pHRR and TSP. The analysis of the char residue showed that APP and KC–Fe have a synergistic effect by further improving the quality of the char residue, thus suppressing the release of volatiles. The KC–Fe-derived iron oxide exhibited a catalytic charring effect with the polyphosphoric acid and metaphosphoric acid produced by APP. A dense char layer structure with the ability to withstand heat and oxygen was formed. Therefore, a thermal stable char layer derived from current flame-retardant intumescent coatings protects the underlying steel in the fire. 

## Figures and Tables

**Table 1:** Formulation of coatings.

Sample	Waterborne Epoxy Resin/g	Curing Agent/g	KC–Fe/g	APP/g
Pure EP	10.71	4.29	-	-
EP/30APP	7.50	3.00	-	4.50
EP/30KC–Fe	7.50	3.00	4.50	-
EP/30APP–KC–Fe (5:1)	7.50	3.00	0.75	3.75
EP/30 APP–KC–Fe (2:1)	7.50	3.00	1.50	3.00
EP/30 APP–KC–Fe (1:1)	7.50	3.00	2.25	2.25

**Table 2:** Thermo-gravimetric analysis (TGA) and DTG data of FR-EP in an N_2_ atmosphere.

Sample	*T*_20wt%_ (°C)	*T*_max_ (°C)	C_800_ (%)
EP	352	389	11.3
EP/30APP	317	325	30.6
EP/30KC–Fe	306	337	28.6
EP/30APP–KC–Fe (1:1)	304	335	36.1
EP/30APP–KC–Fe (2:1)	308	327	35.1
EP/30APP–P–KC (5:1)	317	326	32.1

**Table 3:** Limited oxygen index (LOI) and UL-94 test results of different samples.

Sample	LOI/%	UL-94
EP	18.6	No rating
EP/30APP	30.2	No rating
EP/30KC–Fe	22.8	No rating
EP/30 APP–KC–Fe (1:1)	24.3	V-2
EP/30 APP–KC–Fe (2:1)	29.5	V-1
EP/30 APP–KC–Fe (5:1)	27.3	V-1

**Table 4:** Cone calorimetry data for the pure EP and FR-EP.

Sample	pHRR (kW/m^2^)	THR (MJ/m^2^)	COP (g/s)	TSP (m^2^/kg)
EP	434.5	19.3	0.0146	3.7990
EP/30APP	300.9	11.4	0.0174	2.5270
EP/30KC–Fe	299.4	12.0	0.0039	1.5406
EP/30APP–KC–Fe (1:1)	253.8	11.4	0.0063	2.6448
EP/30 APP–KC–Fe (2:1)	213.2	10.9	0.0058	2.0988
EP/30 APP–KC–Fe (5:1)	215.4	11.1	0.0059	2.3044
